# Development and validation of a prediction equation for body fat percentage from measured BMI: a supervised machine learning approach

**DOI:** 10.1038/s41598-023-33914-5

**Published:** 2023-05-17

**Authors:** Shiming Xu, Roch A. Nianogo, Seema Jaga, Onyebuchi A. Arah

**Affiliations:** 1 Department of Epidemiology, UCLA Fielding School of Public Health, 650 Charles E. Young Drive South, Los Angeles, CA 90095-1772 USA; 2 California Center for Population Research (CCPR), UCLA, Los Angeles, CA USA; 3 Largo Medical Center, Largo, USA; 4 Department of Statistics and Data Science, UCLA, Los Angeles, CA USA; 5 Department of Public Health, Research Unit for Epidemiology, Aarhus University, Aarhus, Denmark

**Keywords:** Cardiology, Diseases, Health care, Risk factors

## Abstract

Body mass index is a widely used but poor predictor of adiposity in populations with excessive fat-free mass. Rigorous predictive models validated specifically in a nationally representative sample of the US population and that could be used for calibration purposes are needed. The objective of this study was to develop and validate prediction equations of body fat percentage obtained from Dual Energy X-ray Absorptiometry using body mass index (BMI) and socio-demographics. We used the National Health and Nutrition Examination Survey (NHANES) data from 5931 and 2340 adults aged 20 to 69 in 1999–2002 and 2003–2006, respectively. A supervised machine learning using ordinary least squares and a validation set approach were used to develop and select best models based on R^2^ and root mean square error. We compared our findings with other published models and utilized our best models to assess the amount of bias in the association between predicted body fat and elevated low-density lipoprotein (LDL). Three models included BMI, BMI^2^, age, gender, education, income, and interaction terms and produced R-squared values of 0.87 and yielded the smallest standard errors of estimation. The amount of bias in the association between predicted BF% and elevated LDL from our best model was −0.005. Our models provided strong predictive abilities and low bias compared to most published models. Its strengths rely on its simplicity and its ease of use in low-resource settings.

## Introduction

Obesity is a major public health problem in the United States. More than one third of Americans considered obese in 2018^1^. Since 1972, the presence of obesity has commonly been assessed in adults using the Quetelet index or body mass index (BMI)^2^. Although BMI plays a very important role in clinical and research setting, it is not an ideal indicator of adiposity for predicting obesity and of obesity-related diseases^3^. This is because BMI is not a direct measure of body composition and it poorly predicts adiposity in populations with excessive fat-free mass^4^. Furthermore, the current BMI-based criteria for defining obesity as BMI greater than or equal to 30^1^, has been shown to misclassify obesity in specific groups like the Asian race/ethnicity^5^. Also, the BMI indicator does not differentiate between visceral fat and non-visceral (subcutaneous) fat. This is important since various kinds of body fat can have different influences on the risks of diseases^6^. In addition to BMI, several indicators such as waist circumference (WC), waist-to-hip circumference (WHC), and waist-to-height, have been commonly used in the clinical and research settings and have proven to be better associated with cardiovascular risk factors compared to BMI^7–9^. Another adiposity indicator—the body adiposity index (BAI), unlike BMI, does not need weight measurement in its calculation^10^. Nevertheless, several follow-up studies have failed to prove its superiority compared to BMI^11–14^.

A third group of method of adiposity assessment—bioelectrical impedance analysis (BIA) and dual X-ray absorptiometry (DXA) which are direct measures can be used to assess body composition, but are not readily available in low-resource settings. Nevertheless, while DXA and BIA correlate relatively well in assessing fat mass and percent body fat, the DXA technique appears to be more accurate, especially among individuals who are obese compared to BIA^15–17^. In fact, BIA generally tends to underestimate fat mass and percentage body fat when compared to DXA^15–17^. However, the DXA method is costlier, more invasive (uses low-dose X-ray) and often requires more technical expertise to use compared to BIA^15–17^. Therefore, given that DXA is highly accurate in assessing fat mass and percentage body fat but costlier than BIA, it is imperative to find ways to predict DXA percentage body fat without having to use DXA.

One way to better and cost-effectively predict adiposity is to create an equation model for body fat percentage (BF%) obtained via DXA but using a cost-effective but imperfect measure such as BMI as its main predictor and other covariates. Although BMI has limited predictive abilities (due to measurement error), using BMI in the prediction equation is ideal since it is still the most widely used measure of adiposity, it is easily calculated and is cost-effective. In other words, this would be akin to correcting an imperfect measurement of adiposity using a predictive model. Additionally, the choice of other covariates in the model is also important as it can help improve the predictive abilities of the equation. However, some potential covariates while their addition can improve prediction accuracy, they may not readily be available in low-resource settings and secondary datasets. Several equations relating BF% and BMI and using various covariates including age, sex, handgrip, waist circumference have been published (Tables 1 and S1)^18–21^. These models did not used rigorous predictive methodologies and have not been developed and validated specifically in a nationally representative sample of the US population. More recently though, Stevens et al. published several similar models using the National Health and Nutrition Examination Survey (NHANES) data and rigorous statistical learning methodologies but used BIA in their prediction model^22^—an adiposity measure that may not be readily available in low-resource setting. Examples of variables that can be difficult to obtain and are not always available in publicly available datasets include handgrip, triceps skinfold, subscapular skinfold, and bioelectrical impedance (BIA) measures. The inclusion of such variables in equations can hinder their use, especially in low-resource settings. Parsimonious prediction model for DXA-BF% that (1) are calibrated to a U.S. general population, (2) include variables (e.g. BMI and socio-demographics only) that are easily accessible in low-resource settings and (3) lead to minimal or low bias when using it in association studies in place of the measured DXA BF% and (4) publish their equation and coefficient for wide use are lacking.

**Table 1:** Comparative characteristics of published parsimonious prediction equations.

Studies	DXA-measured BF%	Sample size > 1000	Validated in U.S. Population	Bias assessment	Supervised machine learning	Included variables^a^	Adjusted R-squared	Root mean squared error (RMSE)
Gomez-Ambrosi^19^	✓	✓				A, sex, BMI	0.79	Not reported
Gallagher^18^	✓					Age, sex, BMI	0.86	Not reported
Fukuda^20^	✓					Sex, BMI, handgrip	0.74–0.84	Not reported
Liu^21^	✓	✓				Age, sex, BMI, WC	0.81	Not reported
Stevens^22^	✓	✓	✓		✓	age, BMI, race, height, weight, BIA, triceps skinfold, subscapular skinfold	0.21–0.88	2.37–6.52

BMI, body mass index; BIA, bioelectrical impedance.

^a^The Fukuda and the Stevens models include variables that are not always readily available (e.g. handgrip, BIA).

We set up this study to create such a model by developing and validating a parsimonious model of BF% as measured by DXA and using only BMI and socio-demographics using a supervised machine learning framework in a nationally representative sample of the US. Additionally, this model will be available online for widespread use for scientists and clinicians.

## Methods

### Study population

**Figure 1:**
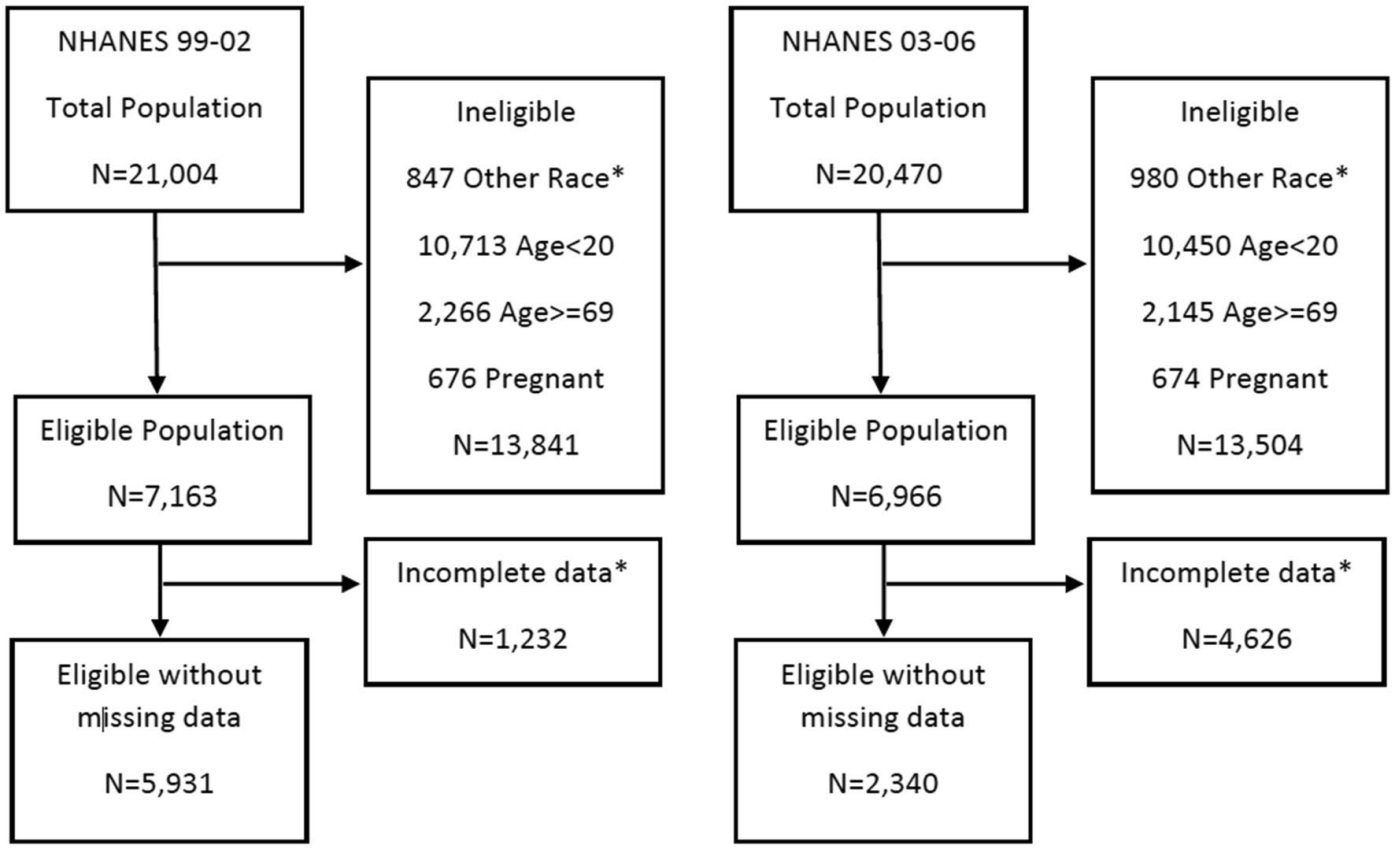
Study Population for NHANES 1999–2006.

Study participants were derived from the National Health and Nutrition Examination Survey (NHANES) 1999–2006^23^. Briefly, NHANES is a nationally representative survey designed to assess the health and nutritional status of adults and children in the United States. The interviews which collect demographic, socioeconomic, dietary, and health-related information as well as the physical examination are conducted on a representative sample of about 5000 individuals each year. In the current study, our sample included all male and non-pregnant female participants aged 20–69 years old of either Hispanic, Caucasian or African descent. Only observations with complete data on all variables studied were included in the analytical sample. The selection resulted in three groups of populations with 5,931 subjects in NHANES 1999–2002, and 2340 subjects in the NHANES 2003–2006^23^ (Fig. 1).

“Other race” means races that do not include black, white, or Hispanic. Incomplete data means having missing data in either BMI, DXA, Age, Gender, Race, Education, or Income data. Data with missing LDL values were kept in NHANES 1999–2002 but excluded in the NHANES 2003–2006 dataset.

### Measurements and socio-demographics

Standing height was measured to the nearest 0.1 cm using a stadiometer. Weight was measured to the nearest 0.1 kg using a digital weight scale^23^. BMI was calculated using the following equation: BMI (Kg/m^2^) = Weight (kg) / height^2^ (m). BF% was estimated by DXA using the Hologic QDR 4500A. The DXA-measured BF% data were only available publicly in NHANES 1999–2006. In NHANES 1999–2002, the BF% was measured in total body area^23^. In NHANES 2003–2006, the BF% was measured at the android area, which was defined as the area between the waist and the mid-point of the lumbar spine and the top of the pelvis^23^. In NHANES 1999–2002, there were two measurements for BF% available: total BF% and subtotal BF% (subtotal = total minus head). We used total BF% instead of subtotal BF% so that our results will be comparable to Liu et al. equation^21^. In NHANES 2003–2006, there were changes in measurements from total/subtotal BF% measurements to android/gynoid BF%. We used android BF% data since android fats are associated with increased risk of obesity-associated diseases such as diabetes mellitus and gynoid fats with a decrease risk of diabetes mellitus^23^. To evaluate the amount of bias that would result from using the predicted BF%, we assessed the association between predicted BF% and low-density lipoprotein (LDL). We chose this relationship because it is known and well established in the literature^24,25^. Measurement techniques of the LDL (mg/dL) sampling was introduced elsewhere^23^. We used the Adult Treatment Panel III Classification of LDL level and defined LDL >  = 130 mg/dL as elevated^26^.

The following socio-demographics were considered for our model: age (year), gender (male vs. female), race (Hispanic vs. White vs. Black), education (high vs. low), and income (high vs. low). The income variable was obtained by categorizing the poverty-income-ratio (PIR) variable in NHANES as either “under the poverty threshold” or “at or above the poverty threshold”. Therefore, low income represented an income under the poverty line and low education represented an education less than high school.

### Statistical analysis

To develop the best parsimonious prediction equation for BF%, we undertook the following steps:

1. *Data preprocessing*: To develop the best prediction model, we used a validation set approach and randomly divided the 1999–2000 NHANES data (n = 5931) into a training and a validation set representing 50% each of the total dataset. Because we were interested in developing a model that could be readily used in low-income settings, we prioritized variables that can easily be obtained at the point of care such as socio-demographics (e.g. age, sex, education, income, race/ethnicity) and BMI. We additionally considered as potential variables the square root transformations for BMI as well as interaction terms between BMI and SES variables and between BMI squared and SES variables. Continuous variables were also visually inspected for normality and were generally found to be normally distributed. We also dichotomized 1) the income variable as high (i.e. at or above poverty line) and low (i.e. below poverty line) and 2) the education variable as high (i.e., equal to or above high school level) and low (i.e. less than high school level). In the candidate prediction models, we therefore included dummy variables for “low education” and that for “low income”.
2. *Model training*: In the training set (i.e., the first random half of the 1999–2000 NHANES dataset, n = 2965), we generated multiple linear models predicting DXA-measured BF% using ordinary least squares and selected the variables using a forward, backward, and stepwise selection procedures. In the selection process, we forced a number of variables to ensure that they will be included in the final model: BMI (kg/m^2^), BMI squared (kg^2^/m^4^), age, gender, race, income, and education variables. The variables that were candidates for selection included interaction terms between SES variables (i.e. education, income) with BMI, and SES variables with BMI squared. The significance levels for entering and exiting the model were set at 0.2. We used adjusted R^2^ as the initial step to select the best models. If several models had the same adjusted R^2^, we considered the one with the lowest Akaike's Information Criteria (AIC) as the better model^27^. In fact, models with higher adjusted R squared, lower AIC and BIC were considered better.
3. *Model validation*: In this validation set (i.e., the second random half of the 1999–2000 NHANES, n = 2966), we first evaluated the performance of the prediction equations developed in the training stage and then also compared the models against other published models. To do so, the prediction accuracy of the prediction models was compared to other published models using the following calculated metrics: standard error of estimation (SEE), paired t-test, means of each predicted BF%, and percentage change of means from the measured values. SEE was calculated as $\sqrt{\frac{\sum_{i=1}^{n}{\left(measure{d}_{i}-predicte{d}_{i}\right)}^{2}}{n}}$. The percentage change of means from the measured values was calculated as $\frac{\left(mean \, of \, predicted \, BF\%\right)-(mean \, of \, measured \, BF\%) }{mean \, of \, true BF\%}*100\%$. Models with the lowest SEE and smallest percentage change of means from the measured values were considered better.
4. *Bias assessment*: We assessed the amount of bias or lack thereof that would occur when using the predicted BF% instead of the unmeasured BF%. To do so, we used the predicted BF% obtained from our equations and the measured BF% and assessed their associations with elevated LDL. The coefficients of the measured and predicted BF% were then compared. The bias was calculated as the difference between the coefficient obtained using the predicted BF% and the coefficient obtained using the measured BF%. We used different samples for this analysis, NHANES 2001–2002 (n = 1383) and NHANES 2003–2006 (n = 2340), to ensure the robustness and reproducibility of our findings. (Fig. S1).

All analyses were conducted using SAS version 9.4 software (Cary, NC) (See “Online Appendix” Sect. 1 for procedure details).

## Results

### Sample characteristics

Descriptive characteristics of NHANES 1999–2002 and 2003–2006 are shown in Table 2. There was 50% and 53% of males in NHANES1999-2002 and 2003–2006, respectively. Most of the population were white (47% vs. 51%), with income at or above poverty line (82% vs. 84%), and with education equal or above high school level (69% vs. 75%) in NHANES 1999–2002 and 2003–2006, respectively.

**Table 2:** Characteristics of the study populations (NHANES 1999–2006).

Variables	NHANES 1999–2002 (n = 5931)		NHANES 2003–2006 (n = 2340)	
	Mean (%)	SE^c^	Mean (%)	SE
Age(year)	43.46	0.18	43.52	0.29
BMI(kg/m^2^)	28.54	0.08	28.23	0.12
Body fat percentage	33.83	0.12	35.01	0.21
LDL (mg/dL)	122.95	0.69	117.14	0.74
Gender				
Male	2991 (50)		1229 (53)	
Female	2940 (50)		1111 (47)	
Races				
Hispanic	1888 (32)		614 (26)	
White	2791 (47)		1188 (51)	
Black	1252 (21)		538 (23)	
Income^*a*^				
High	4865 (82)		1963 (84)	
Low	1066 (18)		377 (16)	
Education^*b*^				
High	4070 (69)		1751 (75)	
Low	1861 (31)		589 (25)	

^a^Income: High means at or above poverty line, low means below poverty line.

^b^Education: High means education is equal to or above high school level, low means education is less than high school level.

^c^SE: Standard error of mean.

### Model training

We first generated 20 models in the training set and selected to the top three models on the basis of adjusted R^2^, AIC and BIC. The best three models selected are presented in Table 3. All selected three models 1, 2 and 3 had an adjusted R squared of 0.87. In addition, model 1, 2 and 3 had an AIC of 7100, 7101 and 7102 respectively. Lastly model 1, 2 and 3 had a BIC of 7102, 7103 and 7104, respectively. The detailed information regarding the coefficients and P-values of the variables for the selected three models were presented in Table S2. Because the significance levels for entering and exiting the model were set at 0.2, some variables included in the selected models had a p-value that was greater than 0.05.

**Table 3:** Variable selections model’s adjusted R^2^, AIC and BIC, NHANES 1999–2002 training set (n = 2965).

Best 3 models	Selected variables	Adjusted R^2^	AIC	BIC
Model 1	BMI*Male, BMI*Age, BMI*Hispanic, BMI*Black, BMI* Low Education, BMI^2*Male, BMI^2*Age, BMI^2*Hispanic, BMI^2*Black, BMI^2*Low Education	**0.8681**	**7100.0**	**7102.3**
Model 2	BMI*Male, BMI*Age, BMI*Hispanic, BMI*Black, BMI^2*Male, BMI^2*age, BMI^2*Hispanic, BMI^2*Black, BMI^2*Low education	0.8680	7100.5	7102.7
Model 3	BMI*Male, BMI*Age, BMI*Hispanic, BMI*Black, BMI*Low Education, BMI^2*Male, BMI^2*Age, BMI^2*Hispanic, BMI^2*Black	0.8680	7101.5	7103.7

The metrics for the top performing model is in Bold.

Forced variables are BMI, BMI^2, gender, race, education, income, age.

Variables available for selection: interaction terms between BMI and SES variables, and BMI squared and SES variables.

Exit and entry levels: 0.2

AIC: Akaike's Information Criteria, BIC: Bayesian Information Criteria.

The best model (model 1) had the following form:

$$\begin{aligned} Model1:BF\% & = - 19.32396 + 2.9479*bmi - 0.03208*bmi^{2} - 7.87425*male \\ & \quad + 4.72996*Hispanic + 4.37595*black + 0.45621*lowIncome \\ & \quad + 2.23965*lowEducation + 0.31443*age - 0.34078*bmi*male \\ & \quad - 0.01385*bmi*age - 0.24071*bmi*hispanic \\ & \quad - 0.42926*bmi*black - 0.18377*bmi*lowEducation \\ & \quad + 0.00657*bmi^{2} *male + 0.00017289*bmi^{2} *age \\ & \quad + 0.00296*bmi^{2} *hispanic + 0.00681*bmi^{2} *black \\ & \quad + 0.0032*bmi^{2} *lowEducation \\ \end{aligned}$$;

### Model validation

As shown in Table 4, our best three models all yielded the smallest value of SEE of 3.29. The developed models 1, 2 and 3 predicted a mean of BF% 33.74, 33.74 and 33.73, respectively. The predicted mean of the developed equations were closest to the measured value (33.75) compared to other models (Model 1 *P* = 0.90, Model 2 *P* = 0.93, Model 3 *P* = 0.85). Likewise, the model by Gomez-Ambrosi produced a predicted mean that were not different from the measured BF% (*P* = 0.17). Moreover, our developed models 1, 2 and 3 produced the smallest percent change in means from the measured BF% (−0.02%, −0.02% and −0.03%, respectively).

**Table 4:** Validating the best three models against previously published models, NHANES 1999–2002 validation set (n = 2966).

Studies	Mean^a^	SEE^b^	Paired t test	*P*-value^d^
			Mean of Difference (%changes^c^)	
Measured BF%	**33.75**	–	–	–
Gallagher^18^	30.27	5.06	−3.47 (−10.28%)	< 0.001
Gomez-Ambrosi^19^	33.64	4.22	−0.11 (−0.33%)	0.17
Liu^21^	31.05	4.37	−2.65 (7.85%)	< 0.001
Model 1	**33.74**	**3.29**	−0.0078 (−0.02%)	0.90
Model 2	**33.74**	**3.29**	−**0.0054 (**−**0.02%)**	**0.93**
Model 3	33.73	**3.29**	−0.0116 (−0.03%)	0.85

The metrics for the top performing model(s) are in Bold.

^a^Mean: Mean of the predicted BF%.

^b^SEE: Standard error of estimation.

^c^%changes: Percentage changes of means from the measured value.

^d^*P*-value: *P*-value for paired t-test.

### Bias assessment

We ran multivariate linear and logistic regressions to assess the risk of elevated LDL utilizing BF%, age, gender, race, education, and income variables in the NHANES 2001–2002 and NHANES 2003–2006, respectively. Measured and predicted BF% were positively associated with elevated LDL status. In the logistic regression, our models produced the smallest bias of -0.005 in NHANES 2001–2002 but did not perform the best in NHANES 2003–2006 (Bias = 0.005 vs. 0.001 in the Gallagher et al. equation and 0.003 in the Gomez-Ambrosi et al. equation) (Table 5). Likewise, in the linear regression, our three models produced the smallest bias of 0.05, 0.06 and 0.06 in NHANES 2001–2002 but did not perform the best in NHANES 2003–2006 (Bias = 0.07 vs. 0.03 for the Gallagher et al. equation) (Table 6).

**Table 5:** Association between percent body fat (measured and predicted) and high low-density lipoprotein (LDL) obtained from logistic regression and bias assessment, NHANES 2001–2006.

Studies	NHANES 2001–2002 (n = 1383)			NHANES 2003–2006 (n = 2340)		
	Odds ratios	95% CI	Bias^a^	Odds ratios	95% CI	Bias^a^
Measured BF%	**1.042**	**1.022–1.062**		**1.02**	**1.010–1.031**	
Gallagher^18^	1.034	1.015–1.054	−0.008	**1.021**	**1.006–1.037**	**0.001**
Gomez-Ambrosi^19^	1.025	1.010–1.041	−0.017	1.017	1.004–1.031	−0.003
Liu^21^	1.053	1.025–1.081	0.01	1.03	1.009–1.052	0.01
Model 1	**1.037**	**1.014–1.060**	**−0.005**	1.026	1.008–1.044	0.005
Model 2	**1.037**	**1.014–1.060**	**−0.005**	1.026	1.007–1.044	0.005
Model 3	**1.037**	**1.014–1.060**	**−0.005**	1.026	1.007–1.044	0.005

The metrics for the top performing model(s) are in Bold.

High LDL is defined as LDL >  = 130 mg/dL.

High LDL was model as a function of BF%, age, race, gender, education, income.

^a^Bias = ln(OR from predicted BF%)—ln(OR from measured BF%).

**Table 6:** Association between measured and predicted percent body Fat obtained from linear regression and bias assessment, NHANES 2001–2006.

Studies	NHANES 2001–2002 (n = 1383)			NHANES 2003–2006 (n = 2340)		
	Coefficients	Bias^a^	*P*-value	Coefficients	Bias^a^	*P*-value
Measured BF%	**0.68**		< 0.001	**0.59**		< 0.001
Gallagher^18^	0.56	−0.12	< 0.001	**0.56**	**−0.03**	< 0.001
Gomez-Ambrosi^19^	0.42	−0.26	< 0.001	0.46	**−0.13**	< 0.001
Liu^21^	0.9	0.22	< 0.001	0.8	**0.21**	< 0.001
Model 1	**0.63**	−0.05	< 0.001	0.66	**0.07**	< 0.001
Model 2	0.62	−0.06	< 0.001	0.66	**0.07**	< 0.001
Model 3	0.62	−0.06	< 0.001	0.66	**0.07**	< 0.001

The metrics for the top performing model(s) are in Bold.

^a^Bias = Coefficient from predicted %BF—Coefficient from measured %BF.

### Ethics approval and consent to participate

Not applicable as this study used public de-identified secondary data. However, NHANES was approved by the CDC ethics review board, and participants provided written informed consent prior to participation. Hence, initial data collection involving humans was conducted in accordance with relevant institutional ethical guidelines.

## Discussion

The purpose of our study was to develop and validate a parsimonious model of BF% using only BMI and socio-demographics factors in a nationally representative sample of the US population. Our best parsimonious model yielded a high adjusted R^2^ of 0.86 and small standard error of estimation of 3.29 and included only BMI, BMI squared, age, gender, race, income, education, and interaction variables. Additionally, our model produced a competitively high adjusted R-squared and low bias in the estimation of the association between BF% and LDL compared to published prediction equations.

Our model can be considered to have strong predictive abilities. In fact, as recommended by Heyward^28^, a good prediction equation needs several characteristics: use of acceptable reference methods; use of large, randomly selected samples (N > 100); high correlation between the reference measures and predicted scores (R^2^ > 0.8); small SEE; cross-validation of equation in samples from an independent population^28^. Our model predicted BF% as measured by DXA—a gold standard reference for measuring adiposity. In addition, we used a large sample sized data of 5,931 from NHANES that was randomly divided in a training and validation sets. The adjusted R-squared was > 0.8. The SEE of the model was 3.29 and thus considered “a very good estimation” since SEE between 3 and 3.5 indicates a very good estimation while a SEE larger than 5 indicates a poor estimation^29^.

Furthermore, we also performed a rigorous supervised statistical learning with a validation set approach and conducted a bias assessment in two separate datasets. The bias assessments in the linear and logistic regression in NHANES 2001–2002 showed that our models yielded a minimally biased association between predicted BF% and LDL and risk of high LDL, respectively (lowest bias). Of importance, is that our models tended to slightly underestimate BF% by −0.02% to −0.03%. This departure between predicted BF% and measured BF% is negligible and could be considered not clinically relevant^30^. Assuming that this underestimation is consistent for the mean BF% in a population, we can always correct and back-calculate the measured BF% from the predicted BF%.

When our models were compared to previously published models by Gallaher (2000), Gomez-Ambrosi (2012) and Liu (2015), the validation results suggested that our models improved the prediction accuracy by around 1% (SEE = 3.29 vs. 5.06, 4.22, 4.37). The improvement of SEE might be due to several factors. First, unlike Liu’s study which was developed in an Asian population and Gomez-ambrosi’s study which was developed in a European population, our model was developed and validated in a representative sample of the US population. Second, our training dataset included a larger number of subjects (n = 2965) which provided more opportunity to detect associations. For example, our model contained the interaction between BMI squared and race, which was not included in any of the other models. Third, Gomez-Ambrosi et al. used BF% estimated from bone density and Siri equation while all the other studies included in our training dataset used DXA-measured BF%. This could be one of the explanations for the differences in validation because our validation dataset also used DXA-measured BF%. In terms of the bias assessment, the results derived from NHANES 2001–2002 suggested that our models had the lowest biases as compared to all the other published models. All the above results indicated our models out-performed other models in the U.S. population. However, the bias assessment conducted in NHANES 2003–2006 showed that the Gallagher’s model did a better job than our models by yielding OR and coefficients that were closer to the measured values as compared to our models. One possible explanation for the results could be the difference in measurements in body fat areas between NHANES 1999–2002 (i.e. total BF%) and NHANES 2003–2006 (i.e. android BF%). Another study conducted by Fukuda et al. included the handgrip variable in their model^20^. We were not able to compare Fukuda’s model in validation and bias assessment because the handgrip data was not available in NHANES 1999–2006. It should be noted that handgrip measurement is difficult to obtain and would make Fukuda et al.’s equation difficult to implement in low-resource settings.

Stevens et al. had recently published a similar study focusing on predicting DXA-measured BF% using NHANES 1999–2006^22^. Nevertheless, there are differences in study design. First and most importantly, Stevens et al. used bioelectrical impedance (BIA) as the main predictor for BF% rather than BMI as in our models. BIA can be difficult to obtain while BMI is commonly utilized in health facilities and is less costly than BIA. Second, Stevens et al. reported that their models that included age, ethnicity, height, weight, BMI and BIA produced an R^2^ of 0.831 in men and 0.864 in women. This was lower than the R^2^ obtained in our models (0.868). Adding triceps skinfold and waist circumference, however, increased the R^2^ of their models to 0.905 in males and 0.883 in female. This comes, however, at the expensive cost of adding hard-to-access measures that can be difficult to obtain in low-resource settings. Third, unlike our models, the 2017 Stevens et al. prediction equation is not easily accessible as Stevens et al. did not readily publish the coefficients in their article and the link provided for their online BF% calculator did not seem to work at the time we tried accessing it (accessed in September, 2017). Fourth, Stevens per se did not conduct a bias assessment as we did in our study. In fact, one important goal in this endeavor was to be able to predict BF% in order to correct for the measurement error due to the utilization of BMI. We could not compare Stevens et al.’s models against our model or assess the amount of bias, or lack thereof, since we did not have the regression coefficients. Lastly, the population age in Steven et al.’s was 8–49 years^22^, while our study population had a much broader age range for adults (20–69).

There are several implications of our BF% equation. First, clinicians could readily calculate each patient’s approximate %BF that would have been otherwise obtained using expensive equipment. Doing so will help better guide decision-making for patient care. Additionally, clinicians can use the model developed here to obtain a better picture of the metabolic health of their patients especially those with a history of hyperlipidemia, diabetes mellitus, or hypertension who come in with low or normal BMI. Second, our prediction model will allow scientists and clinicians to conduct better weight-related research by correcting for the inherent measurement error in BMI. In addition, our model is particularly attractive as it uses easily accessible variables and as such can be utilized by researchers who wish to have a reliable measurement of adiposity but do not have enough resources to obtain it via DXA. As a result, our prediction model could potentially save time and medical expenditure for researchers, doctors, and patients. To facilitate the use of our models and eliminate the need to calculate BF% by hand, we provided an online Percent Body Fat Calculator and a excel calculator to use off-line Supplemental excel document.

Our study was limited in several aspects. First, our model could not be generalized to races beyond Black, White, or Hispanic race/ethnicities because other races consisted of a very small percentage of the total NHANES population. In addition, since underweight (BMI < 18.5) and severe obese (BMI > 40) subjects only comprise small percentages of population (1.6% and 5.8%, respectively) in the training dataset, it was questionable if our model would be accurate for populations with extreme body compositions. Second, there was a change of BF% measurements from total/subtotal BF% in NHANES 1999–2002 to android/gynoid BF% in NHANES 2003–2006, which reduced the accuracy of our bias assessment conducted in NHANES 2003–2006. Third, we focused our prediction model on the prediction of body fat percentage (BF%) obtained via DXA—given its wide use and relative high accuracy in measuring adiposity. We did not build predictive models for other measures of adiposity (e.g. bioelectrical impedance analysis [BIA], body adiposity index [BAI]) and as such our model may not be able to accurately predict adiposity as would be obtained from other measures. Future studies should investigate the relative performance of an adiposity prediction model using several other measurements of adiposity and the same parsimonious model across measurements.

In sum, we developed and validated BF% prediction models with high predictive properties and low bias, tailored to American adults aged 20–69, and which could easily be accessible to clinicians and researchers.

## Supplementary Information

Supplementary Information 1.

Supplementary Information 2.

## Data Availability

The data came from the National Health and Nutrition Examination Survey (NHANES). 1999–2006. It is a publicly available dataset that can be accessed and freely downloaded (with no prior registration needed) here: https://wwwn.cdc.gov/nchs/nhanes/Default.aspx.
