# Supplementary material for: Development and validation of a prediction equation for body fat percentage from measured BMI: a supervised machine learning approach
Source: Sci Rep. 2023 May 17;13:8010. doi: 10.1038/s41598-023-33914-5 (PMC10192430; doi:10.1038/s41598-023-33914-5)
Supplement: Supplementary file 1 — Supplementary Information 1. [file 41598_2023_33914_MOESM1_ESM.docx]

**Supplemental Materials**

**Development and Validation of a Prediction Equation for Body Fat Percentage from Measured BMI: A Supervised Machine Learning Approach**

Shiming Xu*, Roch A. Nianogo*, Seema Jaga, Onyebuchi A. Arah

*co-first authors

**Supplemental File**

**Tables of Contents**

**Tables**

[**Table S1.** Models that predict BF% by BMI from previous studies 2](#_Toc492392107)

[**Table S2**. Model information 3](#_Toc492392109)

**Figures**

[**Figure S1**. DAG illustrating the relationships between and adiposity and elevated LDL risk assessment in NHANES 2001-2006 (image in a separate file) 5](#_Toc492392754)

**Table S1.** Models that predict BF% by BMI from previous studies

| **Studies** |  | **Population** | |  |  | **Models** |  |
| --- | --- | --- | --- | --- | --- | --- | --- |
|  |  |  |  |  |  |  |  |
| Gallagher 2000 | | 671 adults from American and UK | | | | BF%= 64.5 - 848 * (1/BMI) + 0.079 * Age - 16.4 * Male + 0.05 * Male * Age + 39 * Male * (1/BMI) | |
|  |  |  |  |  |  |  |  |
| Gomez_Ambrosi 2012 | | 6123 Caucasians subjects from the university clinic of Navarra, Spain | | | | BF%= -44.988 + 0.503*Age + 10.689*Female + 3.172*BMI-0.026*BMI^2+0.181*BMI*Female-0.02*BMI*Age-0.005*BMI^2*Female+0.00021*BMI^2*Age | |
|  |  |  |  |  |  |  |  |
| Fukuda 2013 | | 107 healthy Caucasian adults from Midwest American | | | | BF% = -5.55 + 10.266 * Sex + 1.55 * BMI - 0.176 * handgrip | |
|  |  |  |  |  |  |  |  |
| Liu 2015 |  | 1059 subjects from Shanghai, China | | | | BF% = -44.65 + 0.437 * BMI + 0.968 * WC + 0.064 * Age + 19.211 * Female - 0.004 * WC^2 - 0.0881398 * Female * WC | |
|  |  |  | | | |  | |
| *BF%, body fat percentage; BF, fat mass; BMI, Body Mass Index; H, height; W, Weight, WC, waist circumference  *Gallagher 2000 has several equations. However, because our study population does not include Asians, so we only used one of the equations that was obtained from non-Asian population. | | | | | | | |

**Table S2**. Model information

| **Model 1** |  |  |  |  |  |
| --- | --- | --- | --- | --- | --- |
| Variables |  |  | Coefficients | | P-value |
| Intercept |  |  | -19.324 |  | <0.0001 |
| BMI |  |  | 2.9479 |  | <0.0001 |
| BMI^2 |  |  | -0.0321 |  | <0.0001 |
| Male |  |  | -7.8743 |  | <0.0001 |
| Hispanic |  |  | 4.7300 |  | 0.0430 |
| Black |  |  | 4.3760 |  | 0.0394 |
| Low Income | |  | 0.4562 |  | 0.0067 |
| Low Education | |  | 2.2397 |  | 0.2372 |
| Age |  |  | 0.3144 |  | <0.0001 |
| BMI*Male |  |  | -0.3408 |  | 0.0036 |
| BMI*Age | |  | -0.0139 |  | 0.0025 |
| BMI*Hispanic | |  | -0.2407 |  | 0.1021 |
| BMI*Black | |  | -0.4293 |  | 0.0013 |
| BMI*Low Education | |  | -0.1838 |  | 0.1129 |
| BMI^2*Male | |  | 0.0066 |  | 0.0003 |
| BMI^2*Age | |  | 0.0002 |  | 0.0162 |
| BMI^2*Hispanic | |  | 0.0030 |  | 0.1889 |
| BMI^2*Black | |  | 0.0068 |  | 0.0007 |
| BMI^2*Low Education | |  | 0.0032 |  | 0.0623 |
|  | |  |  |  |  |
| **Model 2**  Variables |  |  | Coefficients | | P-value |
| Intercept |  |  | -18.957 |  | <0.0001 |
| BMI |  |  | 2.9210 |  | <0.0001 |
| BMI^2 |  |  | -0.0316 |  | <0.0001 |
| Male |  |  | -7.9970 |  | <0.0001 |
| Hispanic |  |  | 5.5006 |  | 0.0162 |
| Black |  |  | 5.0385 |  | 0.0156 |
| Low Income | |  | 0.4645 |  | 0.0057 |
| Low Education | |  | -0.7221 |  | 0.0235 |
| Age |  |  | 0.3228 |  | <0.0001 |
| BMI*Male |  |  | -0.3294 |  | 0.0048 |
| BMI*Age | |  | -0.0144 |  | 0.0017 |
| BMI*Hispanic | |  | -0.2872 |  | 0.0467 |
| BMI*Black | |  | -0.4697 |  | 0.0003 |
| BMI^2*Male | |  | 0.0064 |  | 0.0004 |
| BMI^2*Age | |  | 0.0002 |  | 0.0121 |
| BMI^2*Hispanic | |  | 0.0036 |  | 0.1038 |
| BMI^2*Black | |  | 0.0074 |  | 0.0002 |
| BMI^2*Low Education | |  | 0.0005 |  | 0.1002 |
|  | |  |  |  |  |
| **Model 3**  Variables |  |  | Coefficients | | P-value |
| Intercept |  |  | -18.853 |  | <0.0001 |
| BMI |  |  | 2.9138 |  | <0.0001 |
| BMI^2 |  |  | -0.0315 |  | <0.0001 |
| Male |  |  | -8.1009 |  | <0.0001 |
| Hispanic |  |  | 5.5719 |  | 0.0152 |
| Black |  |  | 5.1265 |  | 0.0140 |
| Low Income | |  | 0.4647 |  | 0.0057 |
| Low Education | |  | -1.0840 |  | 0.0920 |
| Age |  |  | 0.3245 |  | <0.0001 |
| BMI*Male |  |  | -0.3224 |  | 0.0057 |
| BMI*Age | |  | -0.0145 |  | 0.0016 |
| BMI*Hispanic | |  | -0.2936 |  | 0.0422 |
| BMI*Black | |  | -0.4761 |  | 0.0003 |
| BMI*Low Education | |  | 0.0285 |  | 0.1872 |
| BMI^2*Male | |  | 0.0062 |  | 0.0005 |
| BMI^2*Age | |  | 0.0002 |  | 0.0110 |
| BMI^2*Hispanic | |  | 0.0037 |  | 0.0917 |
| BMI^2*Black | |  | 0.0075 |  | 0.0002 |

**Figure S1**. DAG illustrating the relationships between and adiposity and elevated LDL risk assessment in NHANES 2001-2006

X: DXA-measured BF%, represents the gold standard; Y: LDL status; X^*^: BMI predicted BF% (as a measure of body fat but with measurement error); C: SES variables: age, gender, race, income, and educational level
